# A Case of Bullous Pemphigoid Associated With Nivolumab Therapy

**DOI:** 10.7759/cureus.24804

**Published:** 2022-05-07

**Authors:** Nico Gotera, Pablo Weilg, Caio Heleno, Natalia Ferrari-Gabilondo

**Affiliations:** 1 Internal Medicine, MercyOne North Iowa Medical Center, Mason City, USA; 2 Rheumatology, Boston University School of Medicine, Boston, USA; 3 Medical Oncology, Duke University School of Medicine, Durham, USA

**Keywords:** pdl-1 inhibitor, melanoma skin cancer, bullous lesion, bullous dermatoses, nivolumab-related adverse events

## Abstract

This is a case report of new-onset bullous pemphigoid after the initiation of nivolumab for melanoma. Bullous pemphigoid is a rare immune-mediated adverse effect of nivolumab, with fewer than a hundred cases described. The patient initially developed a rash, which later progressed to respiratory symptoms, prompting the discontinuation of nivolumab. He was started on oral steroids, which improved his symptoms. However, while being tapered off the steroids, his rash reoccurred with the development of bullous pemphigoid. The diagnosis was confirmed by increased eosinophils and sub-epidermal vesicle formation compatible with bullous pemphigoid on skin biopsy. The patient was treated with steroids, mycophenolate, doxycycline, and niacinamide with significant improvement in his symptoms.

## Introduction

Nivolumab is an immunoglobin G4 monoclonal antibody that works as a checkpoint inhibitor by preventing programmed cell death 1 ligand from binding, disrupting T cell regulation. This immunotherapy agent is currently FDA-approved and is used in treating multiple malignancies, including melanoma, non-small cell lung cancer, renal cell carcinoma, and various other malignancies [1]. Nivolumab is known to cause various immune-related adverse events (iRAE), with most of those reactions being cutaneously related [2]. One notable rare adverse effect is the development of bullous pemphigoid that can occur weeks to several months after initiation [3]. Currently, the development of this condition is poorly understood and lacks any standardized treatment. We present a case of new-onset bullous pemphigoid in an elderly male with melanoma despite discontinuation of nivolumab approximately eight months prior to presentation.

## Case presentation

An 80-year-old-male with a history of recurrent nodular melanoma of the left medial calf presented with worsening painful, pruritic blisters for two weeks. He was initially diagnosed with stage IIC (pT4b, pN0, M0) nodular melanoma of his left medial calf via wide excisional biopsy with lymph node dissection eighteen months prior to presentation. The initial nodular melanoma was described as Breslow thickness 4.2mm, mitotic rate 6/mm2, ulceration present, and absence of lymphovascular invasion or perineural invasion. Staging positron emission tomography/computed tomography (PET/CT) did not show any abnormal uptake for malignancy. His melanoma progressed after his initial biopsy, with a new subcutaneous nodule growing 2 cm away from the original site four months later. The patient underwent surgical excision of the nodule. The pathology findings were consistent with the previous melanoma biopsy findings, and subsequently, this was concerning for in-transit metastases. PET/CT scan was repeated around this time and did not show any evidence of distant metastatic disease. The patient was started on nivolumab 480 mg every four weeks with the initial plan to continue for one year. He developed a pruritic rash on his chest and upper extremities that appeared 10 days after starting nivolumab. The patient was classified with a grade 2 rash based on the Common Terminology Criteria for Adverse Events (CTCAE) [4].

After three cycles, nivolumab was discontinued due to worsening pruritus, persistent cough, and shortness of breath. Out of initial concern for pneumonitis, a CT of the chest, abdomen, and pelvis with intravenous (IV) contrast was ordered and did not show any signs of pneumonitis, pulmonary embolism, or metastatic disease. In collaboration with dermatology, he was started on oral prednisone with plans to taper off while starting topical triamcinolone over several months. His respiratory symptoms resolved three weeks after initiation of oral prednisone. Three months after initiation of prednisone and seven months after the occurrence of his second nodular melanoma, the patient was found to have a left thigh mass. PET/CT scan showed a solitary focus of this lesion. Magnetic resonance imaging of the brain was ordered to rule out any metastatic disease to the brain and was subsequently negative for metastatic disease. Excisional biopsy of the left thigh mass via surgery was consistent with melanoma. Through collaborative decision-making, the plan was to continue with observation and restage the patient after three months. Seven months after initiation of treatment for his immunotherapy-related rash and while being tapered off oral prednisone, the patient developed painful bilateral blisters of his thighs, which prompted him to be seen in the emergency department. He was treated with IV steroids and then referred back to oncology and dermatology for his current presentation.

His other medical history included actinic keratosis, allergic rhinitis, hypertension, chronic back pain, spinal stenosis, and cardiovascular accidents. He had no other known history of any autoimmune or dermatological conditions. His only other medication was atorvastatin.

**Figure 1:**
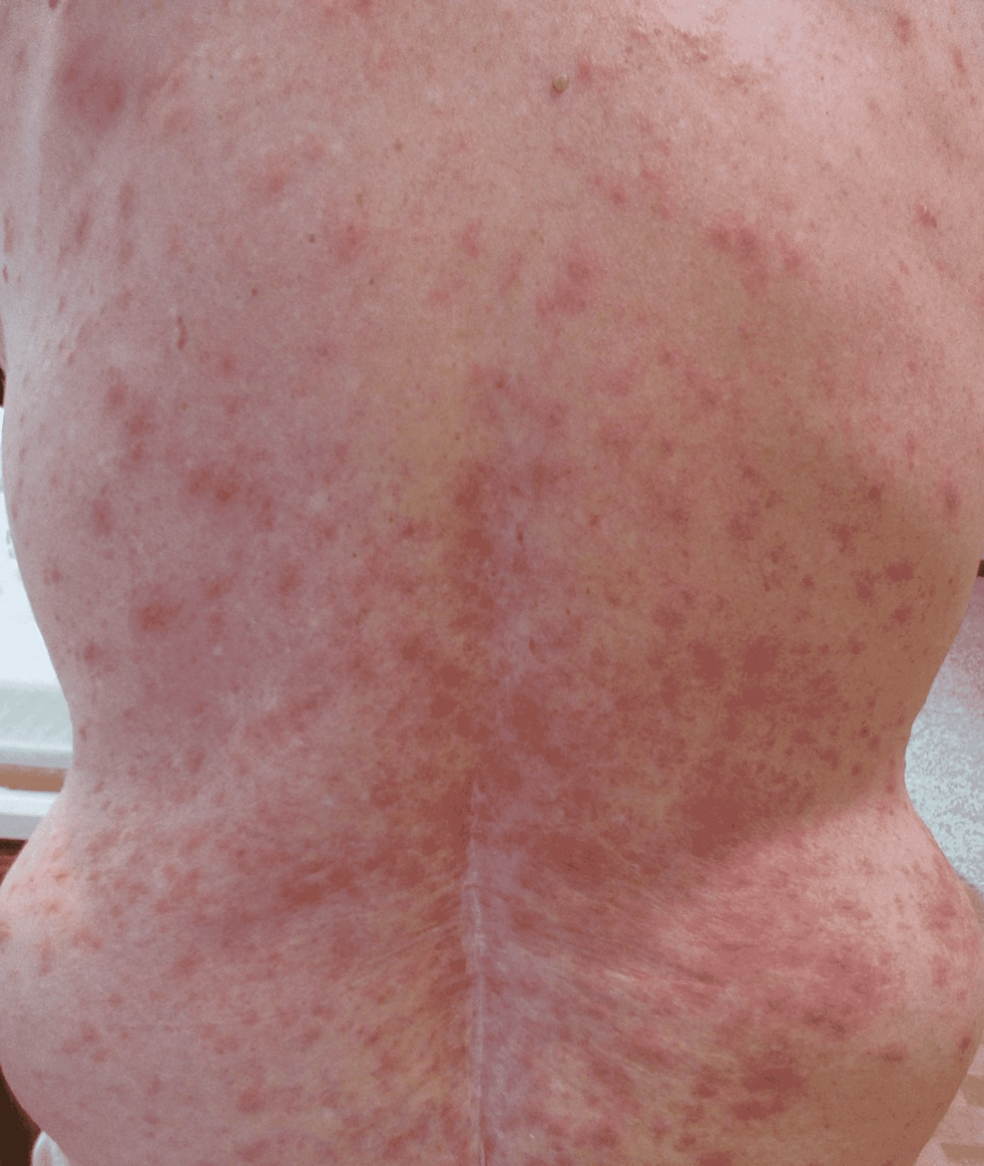
Multiple erythematous background plaques were noted on his posterior trunk.

**Figure 2:**
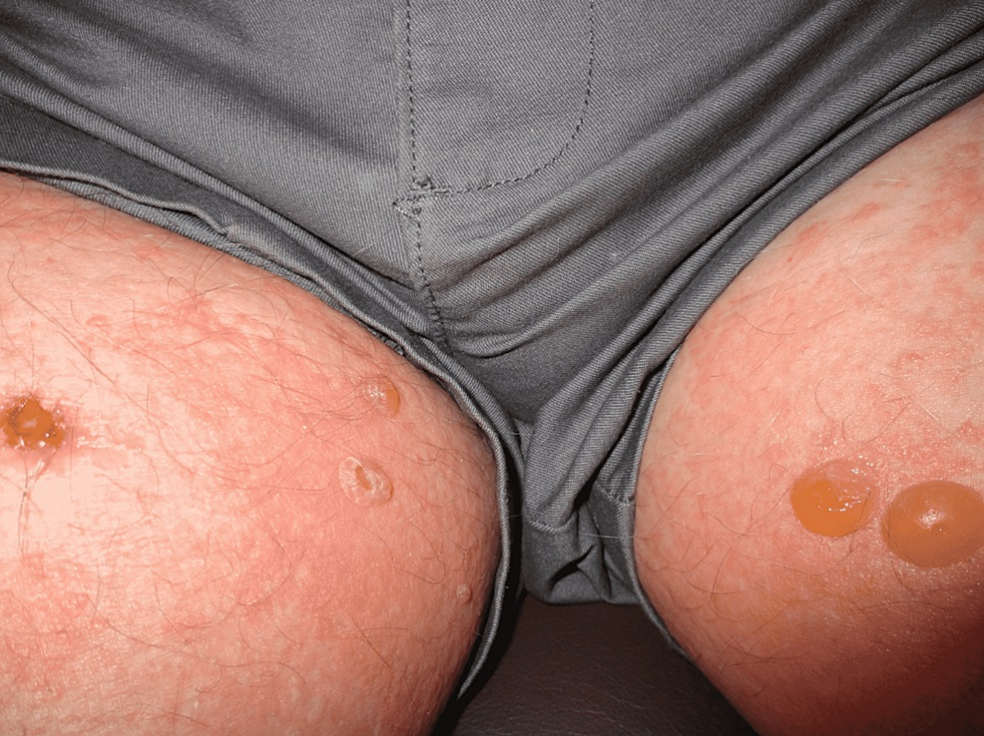
Tense, serous fluid-filled bulla on patient’s bilateral medial thighs.

**Figure 3:**
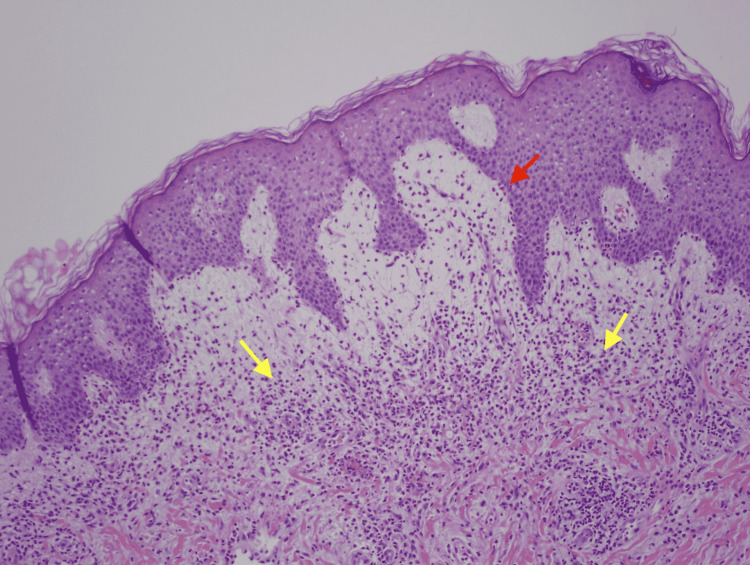
H&E stain showing increased eosinophils and sub-epidermal vesicle formation at 100x magnification. Yellow arrows are pointing at eosinophils—red arrows point at sub-epidermal edema.

**Figure 4:**
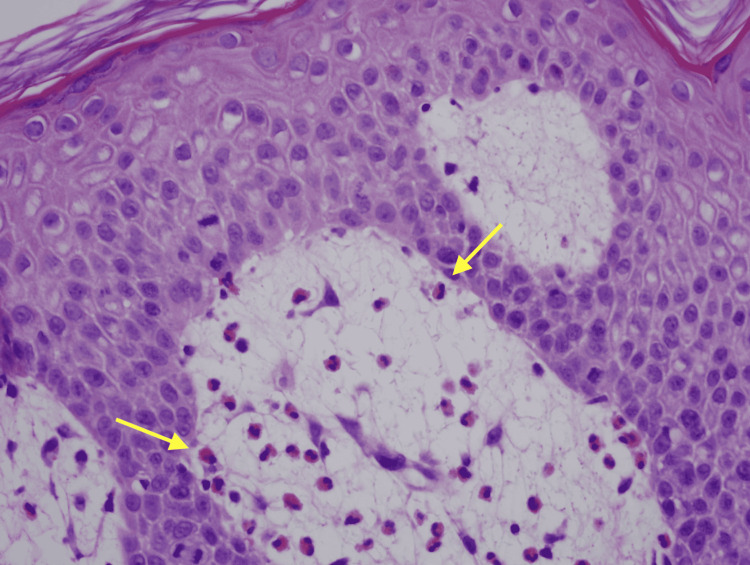
H&E stain showing increased markedly increased eosinophils at 400x magnification. Yellow arrows depict eosinophils.

Physical exam was remarkable for tense, serous fluid-filled bulla on his bilateral medial thighs with erythematous background plaques noted on his trunk/extremities (Figures 1, 2). Initial labs, including a complete metabolic panel and complete blood count, did not show abnormal findings. A repeat PET-CT scan did not show any progression of his melanoma. A punch biopsy of his left thigh showed marked increased eosinophils and sub-epidermal vesicle formation, confirming the diagnosis of bullous pemphigoid (Figures 3, 4). Direct immunofluorescence of his biopsy showed deposition of C3 and IgG consistent with bullous pemphigoid.

**Figure 5:**
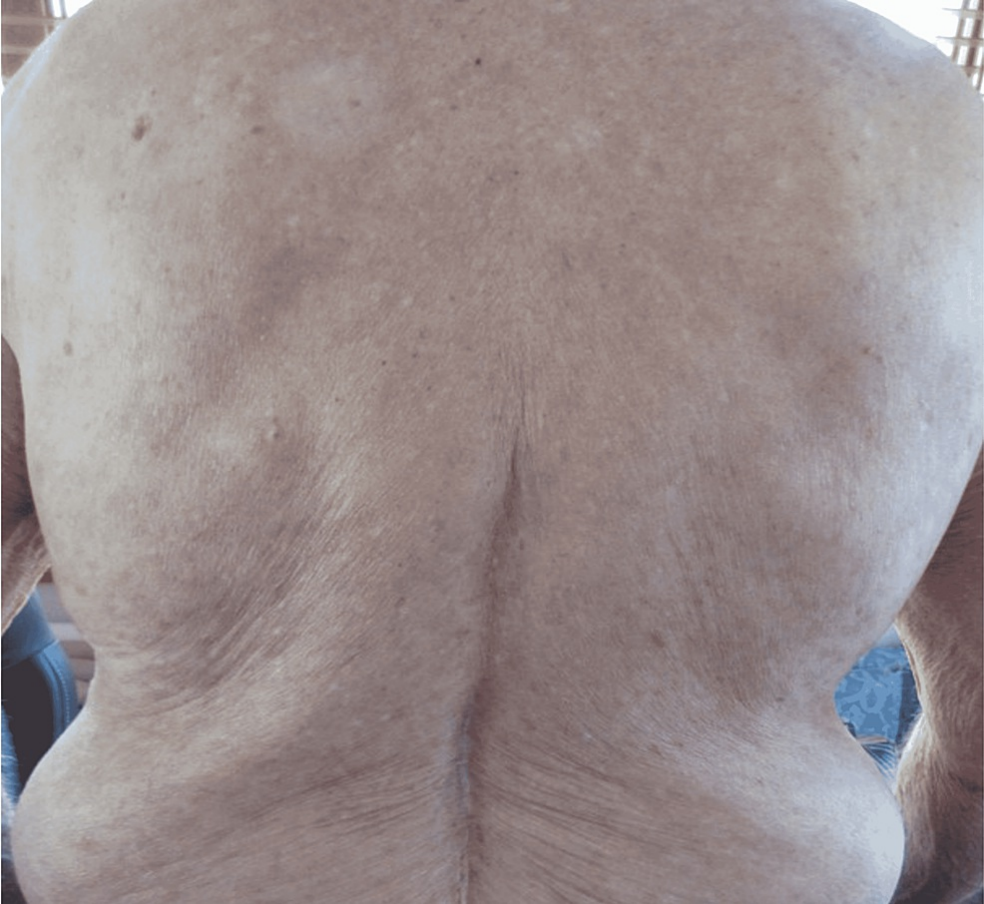
Posterior trunk one month after initiation of treatment with oral prednisone, doxycycline, niacinamide, and mycophenolate.

**Figure 6:**
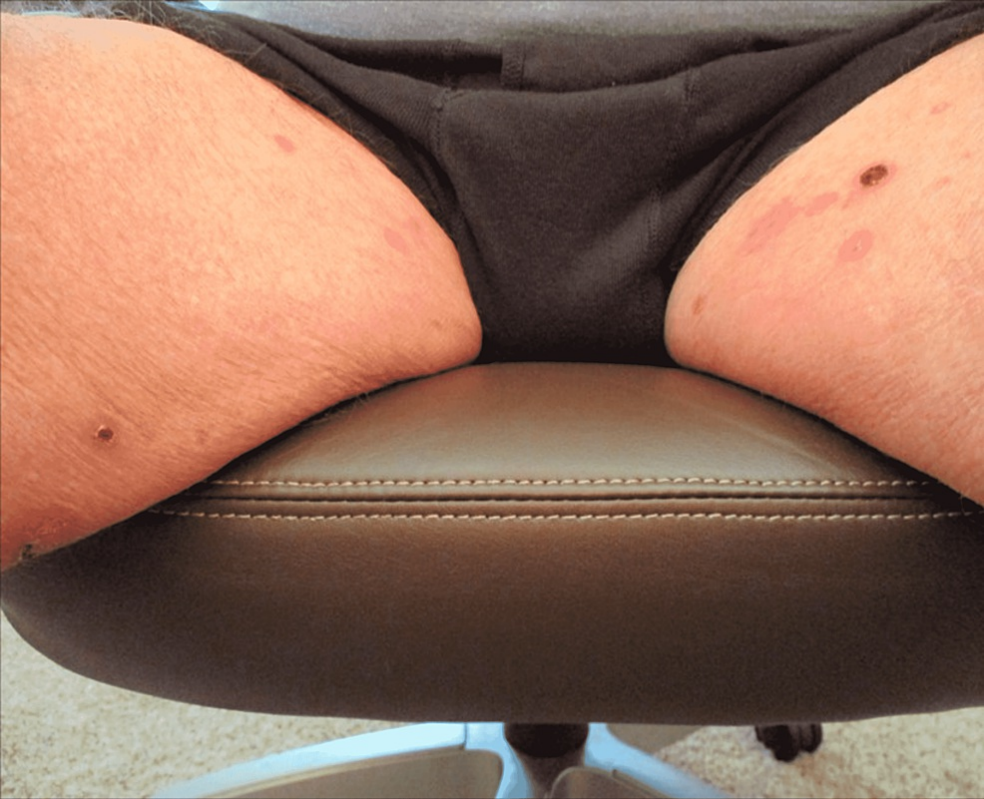
Bilateral lower extremity one month after initiation of treatment with oral prednisone, doxycycline, niacinamide, and mycophenolate.

The patient was classified as a grade 2 bullous dermatitis based on the CTCAE [4]. Through collaboration with dermatology, the patient was started on oral prednisone 60mg daily, doxycycline 100mg twice a day, and niacinamide 500mg three times a day. He was later started on mycophenolate 1 gm twice a day two weeks later after his rash symptoms persisted. One month follow-up after the initial initiation of therapy showed improvement and control of his rash symptoms (Figures 5, 6). Follow-up CT of the chest, abdomen, and pelvis for restaging his melanoma did not show any evidence of metastatic disease.

## Discussion

Nivolumab is typically associated with autoimmune-related adverse effects, given that its mechanism of action promotes T-cell activity. Typically, the most common autoimmune-related adverse effects are gastrointestinal and cutaneous [1]. Other potential rare adverse effects of nivolumab include pneumonitis, endocrinopathies, and myocarditis [1]. Approximately 30-40% of nivolumab-associated adverse effects are cutaneous, including pruritus, maculopapular eruptions, and vitiligo [3]. However, bullous pemphigoid is a rare but acknowledged adverse effect that has been observed in the literature. A MEDLINE search of PubMed for “bullous pemphigoid” and “nivolumab” showed approximately fifty-three patients had developed bullous pemphigoid after initiation of nivolumab in twenty-five case reports and three separate case series [3,5,6]. This did not include any articles that broadly spoke about the class of PD-1 inhibitors causing bullous pemphigoid. The mechanism for why bullous pemphigoid occurs after initiation of nivolumab is not well understood. Theories for the pathogenesis of bullous pemphigoid include the possibility of shared antigens between the cutaneous basement membrane and tumor cells, PD-1 inhibition unmasking bullous pemphigoid in an individual, and PD-1 blockade activating B-cells while inhibiting immunosuppressive B regulatory cells [7].

Bullous pemphigoid is generally caused by idiopathic, infection or drug-related triggers, or malignancy-related causes via paraneoplastic syndromes. Pruritis typically precedes the development of bullous pemphigoid by several months [3]. This can even be more challenging in the non-bullous variant of pemphigoid. Diagnosing nivolumab as the etiology for bullous pemphigoid for our patients can be challenging due to various reasons. Idiopathic causes often occur in elderly males after the age of sixty. However, our patient had no other prior dermatological or autoimmune conditions prior to the onset of symptoms of bullous pemphigoid.

Furthermore, our patient did not have any other new drugs besides nivolumab or recent infections as potential inciting factors for bullous pemphigoid. Finally, a PET-CT scan obtained around the time of diagnosis of bullous pemphigoid suggests against the progression of malignancy or development of a paraneoplastic syndrome. Paraneoplastic bullous pemphigoid is typically associated with B-cell lymphoproliferative disorders and rarely internal malignancies [8]. Furthermore, the association between bullous pemphigoid as a progression of malignancy remains controversial and questionable [3]. Previous case reports and case series describing the timing of the development of bullous pemphigoid after initiation of nivolumab varied from a median of four to eight months, with outliers occurring eighteen months after initiation [3,5,6]. Our case overlapped with the presentation of bullous pemphigoid and initiation of nivolumab occurring eleven months after initiation. One proposed reason why bullous pemphigoid occurs months after initiation of nivolumab is the long average life of PD-1 inhibitors and the persistence of immune response with these agents [9]. The reported average half-life of nivolumab is approximately twenty-seven days with a steady-state of twelve weeks [10]. Given the timing and the lack of other inciting factors, the most likely reason why our patient developed bullous pemphigoid was nivolumab.

From the MEDLINE search of PubMed, it is worth noting that most of the case reports and case series detailed bullous pemphigoid occurring while receiving nivolumab [3,5,6]. Three separate articles detail the development of bullous pemphigoid after discontinuation of nivolumab [3,9,11]. Our case fits into the latter category of developing bullous pemphigoid following discontinuation of nivolumab. Interestingly, the bullous pemphigoid also occurred while being tapered off oral prednisone for his initial pruritic rash. One should be aware that bullous pemphigoid can develop while the patient is receiving, after completion of, or discontinuation of nivolumab.

Our case also notes that having a collaborative effort from a multidisciplinary team, including oncology and dermatology, to manage and treat bullous pemphigoid. There is currently no standardized treatment for nivolumab-induced bullous pemphigoid [3]. However, case reports describe using topical and oral steroids to manage its symptoms. Having dermatology, in this case, was important for initiating the patient on adjunctive therapies for bullous pemphigoid, including doxycycline, niacinamide, and mycophenolate [12]. These agents are reported in studies and case reports for the general manager for bullous pemphigoid. Few studies report the use of doxycycline and niacinamide in managing its symptoms, but mycophenolate has not been studied in particular for nivolumab-induced bullous pemphigoid [3,11]. This step was necessary to stabilize the patient’s rash and prevent reoccurrence one month after initiation of treatment.

## Conclusions

Nivolumab is an immunotherapy agent used in the management of multiple malignancies. Immune-related cutaneous adverse effects are common in nivolumab, but it is important to note that bullous pemphigoid is a rare cutaneous adverse effect that can occur months after initiation. One should be aware that any patient on nivolumab can develop several side effects, such as bullous pemphigoid in our case, despite nivolumab being discontinued or while already on steroid treatment for mild cutaneous adverse effects. Furthermore, a multidisciplinary approach to dermatology and oncology is vital in managing the symptoms of bullous pemphigoid.
